# Outcomes of a Peer Support Program in Multiple Sclerosis in an Australian Community Cohort: A Prospective Study

**DOI:** 10.1155/2013/429171

**Published:** 2013-12-05

**Authors:** Louisa Ng, Bhasker Amatya, Fary Khan

**Affiliations:** ^1^Department of Rehabilitation Medicine, Royal Melbourne Hospital, 34-54 Poplar Road, Parkville, VIC 3052, Australia; ^2^Department of Medicine, Dentistry & Health Sciences, University of Melbourne, Parkville, VIC 3050, Australia; ^3^School of Public Health and Preventive Medicine, Monash University, Clayton, VIC 3800, Australia

## Abstract

*Background/Objectives*. This pilot study evaluated the impact of a peer support program on improving multiple sclerosis (MS) related psychological functions (depression, anxiety, and stress) and enhancing quality of life. *Methodology*. Participants (*n* = 33) were recruited prospectively and received an 8-week group face-to-face peer support program. Assessments were at baseline (T1), 6 weeks after program (T2), and 12 months after program (T3), using validated questionnaires: Depression Anxiety Stress Scale (DASS), McGill Quality of Life (MQOL), and Brief COPE. *Results*. Participants' mean age was 52; the majority were female (64%) and married (64%). Median time since MS diagnosis was 16 years. At T2, participants reported improved psychological functioning (DASS “depression,” “anxiety,” and “stress” subscales, *z* values −2.36, −2.22, and −2.54, moderate effect sizes (*r*) 0.29, 0.28, and 0.32, resp.) and quality of life (MQOL SIS *z* score −2.07, *r* = 0.26) and were less likely to use “self-blame” as a coping mechanism (Brief COPE *z* score −2.37, *r* = 0.29). At T3, the positive improvements in stress (DASS stress subscale *z* score −2.41, *r* = 0.31) and quality of life were maintained (MQOL SIS, *z* score −2.30, *r* = 0.29). There were no adverse effects reported.

## 1. Introduction

Multiple sclerosis (MS) is a chronic demyelinating disease of the central nervous system and one of the most common causes of neurological disability in persons of working age [1]. Persons with MS have a relatively normal life span and live for decades with combinations of deficits, such as physical, cognitive, psychosocial, behavioural, and environmental problems. In 2001, the World Health Organization introduced the International Classification of Functioning, Disability and Health (ICF) [2], which aimed to develop a common language for describing the impact of disease at different levels. Hence, classified according to the ICF, impairments in MS (strength, dysarthria) can result in activity limitation (mobility, self-care) and restriction in societal participation (impact on work, family, and finances). The ICF also includes contextual factors that are divided into “environmental” factors which make up the physical, social, and attitudinal environment in which people live and “personal factors” which include gender, coping style, and social and educational background which may affect the person's experience of living with their condition.

The burden of disease and economic impact of MS upon patients, their caregivers, and on society is substantial. Being diagnosed with a chronic illness, especially one that has no cure or any medical intervention that might stop its progression, is a profound and life-altering event that can result in alterations in physical functioning, loss of control over life circumstances, and subsequent emotional strain [3]. The focus of management thus lies in symptomatic therapy and in achievement of the best quality of life (QOL) for patients and their families. Given the complex, multifactorial nature, and progressiveness of the disabilities in MS, the needs of persons with MS are best met with a coordinated multidisciplinary rehabilitation approach. There are other interventions, however, such as the provision of additional social support, which may complement the process of rehabilitation.

Peer support as a resource has been proposed as an effective means for coping with stressful life experiences and for gaining information and support from others who share a common factor, such as a chronic illness [4, 5]. Mutual identification, shared experiences, and sense of belonging that develops through peer support are thought to impact the psychological outcomes positively [6]. The principal focus of peer support programs is on reducing symptoms and limitations at the level of activity and participation, such as pain and psychological distress and on modifying “personal factors” such as self-efficacy and coping style. These changes are hypothesised to lead directly to changes in health status, which in turn influences health care utilisation [7].

Despite the popularity of peer support programs, there is limited published data on the effectiveness of peer support programs in chronic disease. A systematic review of peer-support programs for people with cancer [8] (*n* = 43 articles) incorporating 5 models of peer support (one-on-one, face-to-face, one-on-one telephone, group face-to-face, group telephone, and group Internet) concluded that there was high satisfaction levels with peer support programs, but the evidence of psychosocial benefit was mixed. Peer mentoring for people with spinal cord injury in a pre-post study (*n* = 37) appeared to enhance self-efficacy beliefs and prevent medical complications [9]. In contrast, Uccelli et al. found that an eight-week peer support program in 44 MS patients did not provide consistent improvement in QOL or depression and further suggested that patients who had better mental health functioning could be at risk for deterioration in support groups [10]. More recently, a study evaluating the impact of social support programs in brain tumour survivors suggested improved emotional well-being and QOL [11] and a small case series (*n* = 7) studying the effectiveness of a six-week face-to-face peer support program in motor neurone disease suggested a trend towards reduced psychological distress [12].

Peer support groups are typically run by volunteers at low cost and have become increasingly popular [10]. The existing data favours other neurological/oncological conditions but not MS and there are no studies to date that look at longer-term outcomes of these interventions. Therefore, the objective of this exploratory study is to evaluate the short- and longer-term impacts of an Australian community-based peer support program on improving psychological distress (anxiety, depression, and stress) and participation (QOL) in persons with MS.

## 2. Methods 

### 2.1. Setting and Participants

This prospective longitudinal pre-post study was conducted at the Royal Melbourne Hospital (RMH) and was approved by the Melbourne Health Human Research and Ethics Committee (HREC 2008.209).

A community-based MS group of 101 patients was identified from the RMH MS database (referred to as the RMH from clinics across Victoria). Inclusion criteria included confirmed diagnosis of MS based on McDonald's criteria [13] as assessed by a neurologist, fluency in English, residence within 30 km of the central business district in Victoria (Australia), ability to communicate and ability and willingness to give informed consent, and age of 18 and above. Those who had severe cognitive issues or dementia or unstable medical, neurological, or psychiatric disorders or were bed-bound were excluded.

### 2.2. Data Collection

All eligible participants were contacted by mail and invited to participate in the study. Those who replied affirmatively were contacted by telephone by the primary researcher who explained the study further. Once signed consent was obtained, participants were informed that it could take up to 3 months before they receive a peer support program due to operational issues (space and the limited availability of facilitators) resulting in difficulties with accommodating all patients concurrently.

This study used a repeat measures design, and each participant was prospectively assessed (face-to-face interviews) at baseline and at 6 weeks and 12 months following completion of the peer support program. Two independent researchers received three half-day training sessions in cognitive and functional ability assessments and completed all assessments. They were observed in a pilot process to confirm achievement of an acceptable standard level. They were not in contact with the treating teams nor shared information about participants or assessments. They received separate and different clinical record forms at each interview and did not have access to medical records, program facilitators, or previous assessments.

Data collected included demographic information, measures of depression, anxiety, and stress, QOL, and coping using standardized instruments (see measures). Assessors did not prompt participants but provided rest breaks and assistance to those who have difficulty with completing the questionnaires. All assessments were secured till the time of entry into the database.

### 2.3. Intervention

All participants received an 8-week group face-to-face community-based peer support program called the LifeMoves program. The LifeMoves program is designed to augment a traditional rehabilitation approach (defined as an inpatient, outpatient, home, or community-based rehabilitation programme, delivered by two or more disciplines such as physical therapy, occupational therapy, and speech pathology, in conjunction with physician consultation, and targeted towards improvement at the levels of activity and/or participation) [14] by targeting the social and emotional consequences of a neurological condition, facilitating psychosocial adjustment and empowering participants to openly express their feelings about and seek solutions to their issues and challenges resulting from their condition. Each program involved attending a weekly two-hour session (with rest breaks) for 8 weeks. Participants were divided into five groups based on days they could attend and these groups ran over a period of two years. Whilst each program was based on participant-generated discussions and hence unique, common topics included coming to terms with the change, hope for the future, managing fatigue, community resources, dealing with emotions, relaxation, and communicating with family and friends.

The senior facilitator facilitated in all groups and provided supervision to other peer facilitators during each session to ensure that facilitation standards were met. All facilitators were volunteers with a neurological condition (either stroke or MS) who had attended a previous LifeMoves program personally. Those who appeared to have good facilitation skills would be selected and encouraged by their facilitators to undergo additional training to become a facilitator themselves. Training involved a 3-hour weekly training program for four weeks, where training was structured to orient the peer to program objectives and the promotion of skills that enable the use of experiential knowledge and the peer's unique understanding of the target population. The training program included information on basic fire safety, roles, boundaries and the importance of confidentiality, basic group rules, how to get the first meeting started, peer relationships and peer support skills, values, beliefs, and attitudes, valuing diversity, communication skills such as active listening, questioning, and summarising, self-care, and integrating experiences and suggestions for resolving questions that may arise during group meetings. All peer facilitators had facilitated in at least one previous LifeMoves program whilst the senior facilitator had been facilitating in such programs for 6 years and was also the coordinator of the program. A priori compliance for session attendance was set at 60% and documented by the facilitator. Participants who attended a minimum of five sessions were classed as “completers.” Adverse effects of the program were recorded and a dedicated phone number was made available to all study participants five working days a week to address any questions or concerns.

### 2.4. Measures

At the time of recruitment, baseline assessments were completed, which included sociodemographic (such as age, gender, and marital status) and clinical information (type of MS, antidepressant medications). The ICF was used as a conceptual basis for choice of outcome measurement. Outcomes were divided into those that described the impact of disease on body structure and function (emotional functions—anxiety, depression, and stress), activity and participation (QOL), and contextual factors (coping mechanisms).

*Depression Anxiety Stress Scale (DASS) [15]*. It is a 21-item instrument, consisting of three 7-item self-report scales that have been designed to measure the negative emotional states of depression, anxiety, and stress. Participants rate the extent to which they experienced each state over the past week on a 4-point Likert rating scale. It has acceptable to excellent internal consistency and concurrent validity. Clinical range, based on normative data, is defined as scores of 16.57 or above for stress, 12.75 or above for anxiety, and 9.26 or above for depression.

*McGill Quality of Life Questionnaire (MQOL) [16]*. It is a valid and reliable 16-item questionnaire, with each question rated from 0 (not at all) to 10 (extremely). There are five domains, two of which are health related (physical well-being, physical symptoms), and three are nonhealth related (existential well-being, psychological symptoms, and support). For each domain, the score is the mean of the values of the relative items. A total rate is obtained as the mean value of the scores of the five domains. In addition, the participant is asked to indicate his/her self-perceived QOL in the past two days in a single-item scale (MQOL-SIS), rated from 0 (very bad) to 10 (excellent).

*Brief COPE [17]*. This is coping inventory of 14 subscales (active coping, planning, positive reframing, acceptance, humour, religion, using emotional support, using instrumental support, self-distraction, denial, venting, substance use, behavioural disengagement, and self-blame). Each subscale has two items. It has good reliability and validity and is a brief measure that assesses several responses known to be relevant to effective and ineffective coping.

### 2.5. Statistical Analysis

A series of descriptive analyses (*n*, %) were conducted on patient demographics and disease characteristics data. The following analyses were conducted on DASS (primary outcome), MQOL, and Brief COPE: given the skewed distribution, analyses were conducted using nonparametric tests (Wilcoxon signed rank tests), comparing the pre- and posttreatment scores, with the baseline score. Effect size statistics (*r*) were calculated and assessed against Cohen's criteria (0.1 = small, 0.3 = moderate, and 0.5 = large effect) [18]. Statistical package for social sciences (SPSS), v. 18.0 (SPSS Inc., Chicago, IL, USA) was used for analysis.

## 3. Results

The sociodemographic and disease characteristics of study participants (*n* = 33) are shown in Table 1. The mean age of the participants was 52; the majority were female (64%) and married (64%). Median time since MS diagnosis was 16 years and a quarter of the participants remained in the workforce. The majority (88%) of the participants were on antidepressants and 21 (64%) were already receiving counselling from a health professional or attending a support group. A significant number (*n* = 20, 61%) had also previously received multidisciplinary rehabilitation. A third of participants (*n* = 10) reported high levels of depression (DASS). Levels of anxiety (DASS) were similarly high with a third reporting moderate-to-severe anxiety.

**Figure 1:**
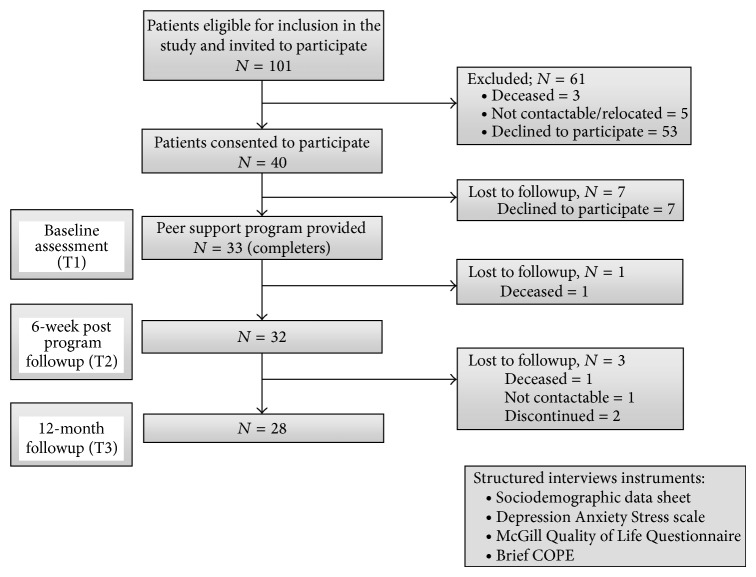
Flow chart of recruitment process.

Of the forty participants who consented to the study, thirty-three attended a minimum of 60% of the sessions. Of the seven “noncompleters,” four stopped attending after three sessions as they shared a car ride to the programme and this was no longer available. All seven “noncompleting” participants were lost to followup at the end of the program (declined to respond to follow-up interview as they felt they had not attended many sessions). A further participant was lost to followup at 6 weeks (deceased) and four more at 12-month followup (1 deceased, 1 not contactable, and 2 discontinued due to illness) (Figure 1). There were no reported adverse effects of peer support.

*Short-Term Subjective Outcomes (Table 2). *At the 6-week followup after the end of the 8-week intervention, participants showed statistically significant improvement in psychological functioning (DASS “depression”, “anxiety,” and “stress” subscales, *z* values −2.36, −2.22, and −2.54, resp., with moderate effect sizes 0.29, 0.28, and 0.32, resp.). As for QOL, MQOL SIS (overall health item) was significantly improved at T2 (*Z* score −2.07, *r* = 0.26) and based on the Brief COPE, participants were less likely to use self-blame as a coping mechanism at T2 (*z* score −2.37, *r* = 0.29). Overall, the participants tended to prefer problem-focused coping strategies to emotion-focused coping strategies. 

*Longer-Term Subjective Outcomes (Table 2)*. At the 12-month followup, improvements in stress, as measured by DASS, remained statistically significant (*z* score −2.41, *r* = 0.31), but there were no differences in the other self-reported psychological functioning domains of DASS (anxiety and depression). Improvements in the overall QOL (MQOL SIS) also remained significant (*z* score −2.30, *r* = 0.29). No changes were seen in coping mechanisms based on the Brief COPE.

## 4. Discussion

To our knowledge, this is the first positive report of short- and longer-term effectiveness of peer support in the MS population in an Australian community cohort. The findings from this prospective pilot study suggest that peer support programs for MS patients targeting specific behaviour, coping strategies, and self-management techniques improved psychological functioning and QOL. The magnitude of improvement in psychological function peaked at the 6-week post treatment period and was in part maintained at the 12-month review. The participants in this study were similar to those in other studies in terms of age, gender, disease severity, and treatment [10, 14].

The positive effects of peer support programs on psychological function are consistent with reports in other neurological and cancer groups [11, 12, 19], but contrasts with previous findings by Uccelli et al. [10] who found that peer support groups did not result in consistent improvement in QOL or depression in patients with MS. Significantly, this Australian cohort had already received high levels of treatment for their psychological distress, with 88% of the participants on antidepressant medication, 64% already receiving counselling from a health professional or attending some form of support group, and 61% having previously received multidisciplinary rehabilitation. Uccelli et al. [10] speculated that the reason support groups fail to produce improvement is they do not meet the needs of the participants. This study appears to support his hypothesis; in a cohort of participants whose needs have already been addressed through medical and rehabilitative means, one would then see the benefits of peer support. Hence, as previously suggested, peer support programs complement and augment a traditional rehabilitation approach but do not replace it. Also of note is that despite having received rehabilitation/other treatment, a significant proportion of people with MS experienced ongoing psychosocial morbidity, which appeared amenable to intervention in the form of peer support.

Other literature has suggested that peer support can have beneficial effects on participants who value camaraderie and comparison [20]. An important advantage to exposure to peer support is getting advice on practical aspects on managing their neurological condition (disability management, home adaptations). Many also enjoy the sense of camaraderie from just being with other people who understand. Seeing others coping well with the condition can provide hope, while downward comparison with those worse off can also make people feel better about their own situation [20]. Levels of involvement may change over time as people struggle with their changing needs and fears [20]. Whilst some of the effects (stress, QOL) of a peer support program in this study appeared to have lasted 12 months, other effects (depression, anxiety) did not appear to last. This is consistent with other literature [20]. Hence, it might be important for peer support to be offered at regular intervals, especially given the changes in functional abilities that persons with MS may experience, although this needs to be explored.

Outcome measurement in MS/peer support research is challenging and varies in different studies [12, 21]. Quality of life in particular is a broad concept, and not easily incorporated in a single outcome measurement. The measurement of QOL is influenced by many factors such as physical, psychological, and cognitive disabilities and participatory limitations. Generic measures commonly used in practice may not include all domains relevant for persons with MS and may not be sensitive to change. However, although MS-specific QOL measures (such as the MSQOL scale) exist and are widely used, the lack of inclusion of existential elements (perception of purpose, meaning of life, and capacity for personal growth) relevant for people with MS and often addressed during peer support programs determined the choice of MQOL as the QOL outcome measurement for this particular study.

This study has some potential limitations. Firstly, this is a longitudinal observational study (no control group), which limits the ability to draw casual relationships between the support program and outcomes. However, it was ethically difficult to withhold the intervention since peer support is generally seen as a positive intervention by patients. Secondly, participants were a selective cohort listed on a database (voluntary participation) held at single tertiary institution, which limits generalisability of findings. Specifically, all participants lived within a 30 km radius due to travel requirements, limiting generalisability to geographically isolated patients (such as rural and remote areas of Australia). Thirdly, participants in this study were complex in terms of disease severity, symptoms, and psychosocial situations (reflective of clinical practice). The likelihood of progressive functional decline, the difficulty in psychological adjustment due to constantly changing disability, and uncertain prognosis created challenges that in turn influenced the type and intensity of the intervention provided. This study, however, neither attempt to control these factors nor control for group effects (such as if a group interacted more positively than another group). The facilitators worked with each participant within a group setting to determine topics for discussion and provide an appropriately tailored peer support program. Lastly, compliance and attendance in sessions were challenging. Because of their MS, a number of patients could not drive resulting in a high reliance on others for transport and hence a relatively high level of attrition. However, most patients who started the program attended a minimum of 60% of the sessions. This suggests that alternate models of peer support that could complement, extend, or even replace face-to-face programs should be further explored. This study was conducted in real-life setting with limited resources and funding. A more rigorous study with a control group, larger MS cohorts in different settings, and single-group-based social support program (providing intervention simultaneously to all participants) would be helpful to establish the generalisability and validity of these results.

Multiple sclerosis has profound impact on function and participation. Physical and psychological morbidity in MS is well documented and the effectiveness of multidisciplinary rehabilitation is well supported [21]. However, this pilot study has shown that peer support programs further improve psychological functioning and QOL with maintenance of benefit for up to 12 months. Peer support programs as an adjunct to multidisciplinary rehabilitation need further evaluation in well-designed clinical trials, over an even longer period of time (2 years or more) and with booster (repeated) peer support intervention. Significant variability within the study sample highlights the need for targeted peer support programs tailored to needs and goals of each person over an extended time period.

## Figures and Tables

**Table 1:** Sociodemographic characteristics of participants (*n* = 33).

Characteristics	*n*, (%) (unless stated different)
Age (years) [mean (SD), range]	51.8 (9.3), 28–66.8
Sex female	21 (63.6)
Marital status	
Married/partner	21 (63.6)
Single/divorced/separated/widow	12 (36.4)
Living with	
Alone	7 (16.3)
Partner/family	36 (83.7)
Employment	8 (24.2)
Disease duration (years) [Md, (IQR)]	16.0 (9.0, 23.0)
MS type	
RR	13 (39.4)
PP	4 (12.1)
SP	16 (48.5)
PR	0 (0.0)
Currently on antidepressant medication	29 (87.9)
Support group attendance	14 (42.4)
Received counselling	7 (21.2)
Counselling source	11 (26.2)
SW	1 (3.0)
Psychologists	4 (12.1)
Psychiatrist	1 (3.0)
Other	1 (3.0)
Previous rehabilitation	20 (60.6)
DASS group: (*n*, %)	
Depression	
Normal/mild	23 (69.7)
Moderate/severe/extreme severe	10 (30.3)
Anxiety	
Normal/mild	23 (69.7)
Moderate/severe/extreme severe	10 (30.3)
Stress	
Normal/mild	28 (84.8)
Moderate/severe/extreme severe	5 (15.2)

DASS: Depression Anxiety Stress Scale; IQR: interquartile range; Md: median; MS: multiple sclerosis; *n*: total number; SD: standard deviation; RR: relapsing remitting; PP: primary progressive; SP: secondary progressive; PR: progressive relapsing.

**Table 2:** Change scores in subscales for measurement scales over time.

Scales	T1 baseline Md (IQR) *n* = 33	T2 6-week Md (IQR) *n* = 32	T3 12-month Md (IQR) *n* = 28	*Z* values		Effect size	
				T1-T2	T1–T3	T1-T2	T1–T3
DASS							
Total (0–126)	24 (13 to 41)	16 (8 to 27.5)	22 (12 to 34.5)	−2.86∗	−1.93	0.35	0.25
Depression (0–42)	10 (4 to 19)	4 (0 to 12)	7 (2 to 14)	−2.36∗	−1.47	0.29	0.19
Anxiety (0–42)	4 (2 to 13)	4 (0 to 7)	4 (2 to 8)	−2.22∗	−1.59	0.28	0.20
Stress (0–42)	12 (5 to 17)	6 (3 to 11)	10 (4 to 14)	−2.54∗	−2.41∗	0.32	0.31
MQOL^#^							
Total (0–160)	91 (78.5 to 111)	94 (81 to 120.5)	96.5 (84.2 to 123.7)	−0.73	−1.06	0.09	0.14
Single-item scale (SIS) (0–10)	6 (4 to 8)	7 (5 to 8)	7 (5 to 8)	−2.07∗	−2.30∗	0.26	0.29
Physical well-being item (0–10)	6 (4 to 8)	6 (4.2 to 8)	7 (5 to 8)	−0.64	−1.16	0.08	0.15
Physical symptoms (0–30)	16 (10.5 to 19)	15 (10.5 to 19.5)	15 (8.5 to 21.7)	−0.35	−0.04	0.04	0.01
Psychological symptoms (0–40)	27 (22 to 37)	31 (24.5 to 36.8)	31 (26 to 38)	−1.20	−1.69	0.15	0.22
Existential well-being (0–60)	37 (30 to 43)	40 (31.8 to 48.5)	39.5 (30.2 to 49.5)	−0.64	−0.91	0.08	0.12
Support (0–20)	15 (11.5 to 17.5)	15 (11.5 to 17.5)	16 (12 to 18.7)	−0.88	−1.19	0.11	0.15
Brief COPE							
Total (28–112)	63 (54.5 to 69.5)	64 (57.2 to 67)	64 (56.2 to 70)	−0.34	−0.18	0.04	0.02
*Problem-focused coping strategies *							
Active coping (2–8)	6 (4.5 to 7)	7 (5 to 8)	6 (5 to 7.7)	−0.31	−0.25	0.04	0.03
Planning (2–8)	6 (4.5 to 7)	6 (5 to 7.7)	6 (5 to 7.7)	−0.52	−0.02	0.06	0.00
Positive reframing (2–8)	6 (4.5 to 7.5)	6 (5 to 7)	6 (4 to 7)	−0.33	−0.72	0.04	0.09
Acceptance (2–8)	6 (5.5 to 8)	6.5 (5.2 to 8)	6 (5 to 7)	−0.37	−0.84	0.05	0.11
Humour (2–8)	4 (3 to 6)	4 (3 to 7)	5 (4 to 6.7)	−0.56	−1.46	0.07	0.19
Religion (2–8)	3 (2 to 5.5)	4 (2 to 6)	3.5 (2 to 4.7)	−1.18	−0.06	0.15	0.01
Using emotional support (2–8)	5 (3 to 7)	5 (4 to 7)	5 (4 to 7)	−0.27	−1.09	0.03	0.14
Using instrumental support (2–8)	4 (3 to 7)	5 (4 to 7)	4.5 (4 to 6)	−0.29	−0.07	0.04	0.01
*Emotion-focused coping strategies *							
Self-distraction (2–8)	5 (4 to 6)	5 (3.2 to 6)	5 (4 to 6)	−1.43	−1.30	0.18	0.17
Denial (2–8)	2 (2 to 3)	2 (2 to 3)	2 (2 to 3.7)	−0.29	−0.15	0.04	0.02
Venting (2–8)	3 (2 to 4)	3 (2 to 4)	4 (3 to 4)	−0.86	−0.02	0.11	0.00
Substance use (2–8)	2 (2 to 2)	2 (2 to 2)	2 (2 to 2)	−0.44	−0.18	0.05	0.02
Behavioural disengagement (2–8)	2 (2 to 3)	2 (2 to 4)	2 (2 to 3)	−0.79	−0.25	0.10	0.03
Self-blame (2–8)	2 (2 to 4.5)	2 (2 to 4)	3 (2 to 4)	−2.37∗	−1.17	0.29	0.15

^*^Correlation significant at 0.05 level (2-tailed).

^#^In scoring MQOL, data were transposed prior to data analysis where necessary (items 1–3 and 5–8), so that a score of “0” always indicated the least desirable and “10” the most desirable situation. For items 1–3, a transposed score of “10” is assigned when the symptom indicated is “none.”

DASS: Depression Anxiety Stress Scale; IQR: interquartile range; Md: median; MQOL: McGill Quality of Life; *n*: total number.
